# The Association of Stage 1 Hypertension, Defined by the 2017 ACC/AHA Guidelines, With Cardiovascular Events Among Rural Women in Liaoning Province, China

**DOI:** 10.3389/fcvm.2021.710500

**Published:** 2021-08-12

**Authors:** Jiake Wu, Weili Duan, Yundi Jiao, SiTong Liu, LiQiang Zheng, YingXian Sun, ZhaoQing Sun

**Affiliations:** ^1^Department of Cardiology, Shengjing Hospital of China Medical University, Shenyang, China; ^2^Department of Clinical Epidemiology, Shengjing Hospital of China Medical University, Shenyang, China; ^3^School of Public Health, Shanghai Jiao Tong University School of Medicine, Shanghai, China; ^4^Department of Cardiology, The First Affiliated Hospital of China Medical University, Shenyang, China

**Keywords:** cardiovascular events, hypertension, rural women, guideline, public health

## Abstract

**Background:** The recent American College of Cardiology/American Heart Association (ACC/AHA) guidelines redefined blood pressure levels 130-139/80-89 mmHg as stage 1 hypertension. However, the association of stage 1 hypertension with cardiovascular disease (CVD) and its age-specific differences among the rural women in Liaoning province remains unclear. It needs to be quantified in considering guideline adoption in China.

**Methods:** In total, 19,374 women aged ≥35 years with complete data and no cardiovascular disease at baseline were followed in a rural community-based prospective cohort study of Liaoning province, China. Follow-up for the new cases of CVD was conducted from the end of the baseline survey to the end of the third follow-up survey (January 1, 2008–December 31, 2017). Adjusted Cox proportional hazards models were applied to estimate the Hazard Ratios (HR) and 95% Confidence Intervals (CI) with the normal blood pressure as a reference.

**Results:** During the median follow-up period of 12.5 years, 1,419 subjects suffered all-cause death, 748 developed CVD, 1,224 participants suffered stroke and 241 had Myocardial Infarction (MI). Compared with normal BP, Stage 1 hypertension had a HR (95% CI) of 1.694 (1.202–2.387) in CVD mortality, 1.575 (1.244–1.994) in the incidence of stroke. The results obtained that the risk of CVD mortality and incidence of stroke was significantly associated with stage 1 hypertension in rural women aged ≥45 years after adjusting for other potential factors. However, in participants aged 35–44 years, stage 1 hypertension was not associated with an increased risk of cardiovascular disease.

**Conclusions:** The newly defined stage 1 hypertension is associated with an increased risk of CVD mortality and also incidence of stroke in the rural women aged ≥45 years population of Liaoning province. This study can be a good reference for health policy makers and clinicians workers to make evidence-based decisions toward lowering burden of cardiovascular disease more efficient, timely measures on prevention and control of stage 1 hypertension in China.

## Introduction

In 2017, the American College of Cardiology/American Heart Association (ACC/AHA) released an updated guideline with a new criteria for hypertension, defining stage 1 hypertension as Systolic Blood Pressure (SBP) as 130 mmHg through 139 mmHg or Diastolic Blood Pressure (DBP) as 80 mmHg through 89 mmHg. People with this BP range are advised to take medication or have lifestyle interventions based on their 10-year atherosclerotic cardiovascular disease risk (1). These recommendations were vital because there was a need to effectively manage hypertension, thereby preventing the prevalence of Cardiovascular Disease (CVD). In addition, the impact of these guidelines on health economics deserves our attention (2). In China, over diagnosis and treatment of stage 1 hypertension may occur in circumstances unrelated to adverse events. Therefore, it becomes important to consider the relationship between stage 1 hypertension and cardiovascular disease risk. Currently, CVD is now the leading cause of death worldwide, accounting for approximately one-third of all deaths, particularly in women. It is also common knowledge that changes in Blood Pressure (BP) in women differ from those in men, a disparity that can be attributed to endocrine changes. Therefore, the onset and treatment of hypertension in women is different from that in men (3). Notably, a nationwide survey was conducted from 2012 to 2015 to assess the prevalence of hypertension in China. The results obtained in the survey indicated that the awareness, treatment, and control rates of hypertension among women was 51.9, 46.6, and 17.7%, respectively (4). In addition, Gu et al. reported that the degree of awareness, treatment, and management of hypertension was significantly lower in women than in men. About one sixth of women live in rural China, shifts in cardiovascular disease in this group have enormous on public health (5). Therefore, to what extent stage 1 hypertension affects CVD warrant careful investigations in rural Chinese women. We aimed to assess the relative risk of CVD associated with stage 1 hypertension and whether the risk was age-specific through a 12.5-year prospective cohort in rural areas of Liaoning Province, China (6).

## Methods

### Study Population

**Figure 1:**
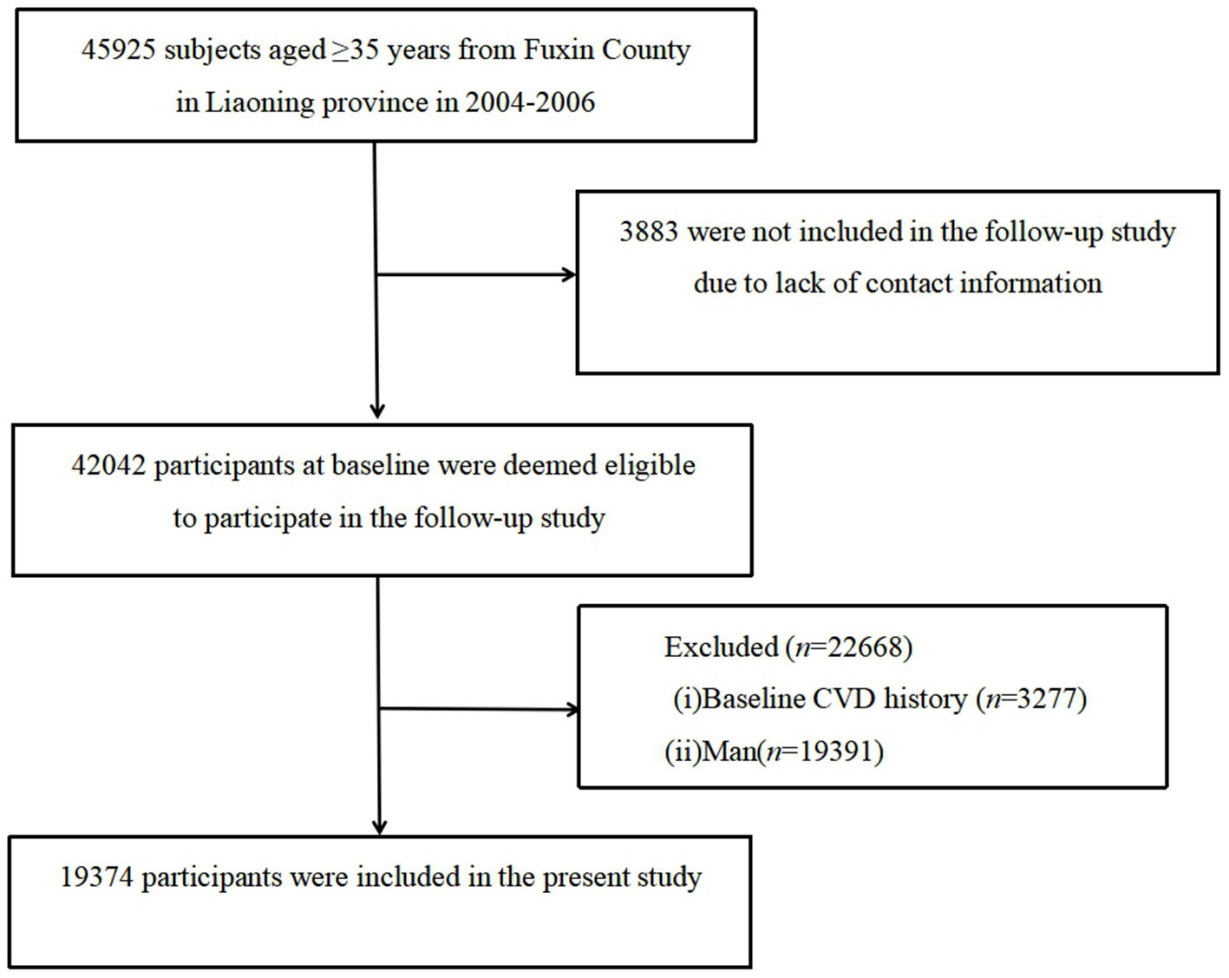
The inclusion and exclusion criteria for the study population.

This was a large-scale epidemiological prospective cohort study. The study involved a randomly stratified, multistage, and cluster-sampling scheme comprising of participants aged ≥35 years recruited from 2004 to 2006. The primary objective of the study was to assess the incidence, prevalence, and risk factors for CVD. The first follow-up was conducted between January and July 2008, the second between July and December 2010, and the third between March and December 2017. Additional cases of adverse events such as mortality, stroke, and Myocardial Infarction (MI), were also recorded from the end of the baseline survey to the end of the third follow-up survey (January 2007–December 2017) (7, 8). Notably, 3,883 out of the 45,925 (8.5%) participants recruited at baseline were excluded from the study because they either lacked contact information or declined to appear during follow-ups, and out of the 42,042 (91.5%) participants were entitled to appeared at least once during the follow-up survey. Among them, a total of 22,668 participants with the following characteristics were excluded: (a) participants suffering from CVD (i.e., myocardial infarction (MI), stroke including arrhythmia, Coronary Heart Disease [CHD] and angina based on self-reports from participants) before baseline or at baseline survey (participant self-reporting) (*n* = 3,277), and (b) males (*n* = 19,391). Finally, 19,374 participants were eligible for further analysis (Figure 1).

### Blood Pressure Measurements

The BP readings were taken by trained healthcare personnel using standard electronic sphygmomanometers (HEM-741C; Omron) according to the guidelines of the American Heart Association (9). The participants were requested to avoid alcohol, cigarettes, hot beverages, and physical activities at least 30 min and to rest for at least 5 min in sitting position before their BP was taken. Three BP measurements were taken per person and the average was used for final analysis (10). Thereafter, the participants were categorized into the following four groups as recommended by the 2017 ACC/AHA guidelines: Normal BP (SBP = <120mmHg and DBP = <80mmHg), elevated BP (SBP = 120–129mmHg and DBP = <80mmHg), stage 1 hypertension (SBP = 130–139mmHg or DBP = 80–89mmHg), and stage 2 hypertension (SBP≥ 140mmHg or DBP≥ 90mmHg or taking antihypertensive medications) (1).

### Data Collection

The demographic data of the participants were derived from standard questionnaire administered by qualified personnel (gender, age, and ethnicity), lifestyle aspects (physical exercise, drinking, and smoking), disease history (stroke, history of hypertension in the family, CHD, diabetes, or hyperlipidemia), and information on anti-hypertensive medications. Furthermore, the participants wore light clothing and had no shoes when measuring height and weight. The Body Mass Index (BMI) was computed weight in kilograms divided by the square of height in meters. In addition, the current smoking status meant smoking at least one cigarette/day for at least 1 year (11), while the consumption of wine, beer, or liquor equivalent to ≥1 glass/day were referred to current drinking status (12). Moreover, physical activity was defined a either low, moderate, or high, depending on the level of physical activity reported by the participant (13). On the other hand, the family history of hypertension meant that at least one parent had hypertension. Additionally, participants were requested to report if they had earlier been diagnosed with a stroke, diabetes/hyperlipidemia, and CHD. Data pertaining to adverse events, and concurrent use of medication was also collected during each follow-up survey.

### Study Outcomes

The primary endpoint was adverse event, which was a composite endpoint that included all-cause mortality, incident MI and stroke, CVD mortality. During the follow-up period, if more than one event occurred in a participant, the first event was considered the endpoint. In the specificity analysis of the endpoints, all-cause mortality, incident MI and stroke, CVD mortality were each considered secondary endpoints. In the secondary endpoint analysis, if a participant had different types of events, the first event of each type was considered the endpoint event. Information about the death was obtained from direct contact with the family and hospital records. In addition, deaths recorded due to cardiovascular diseases were coded from 400 to 444 in accordance to the International Classification of Diseases (ICD), 9th Revision, Clinical Modification (14). MI was defined based on the consensus document of the Joint European Society of Cardiology/American College of Cardiology Committee as a transient increase in laboratory markers specific to myocardial necrosis (CK-MB or troponin T) in combination with ischemic symptoms and/or typical electrocardiographic signs (development of pathologic Q-waves or ST segment elevation or depression) (15). We used the WHO Multinational Monitoring of Trends and Determinants in Cardiovascular Disease criteria to define stroke as rapidly developing signs of focal (or global) disturbance of cerebral function lasting >24 h (unless interrupted by surgery or death) with no apparent non-vascular cause (16). A non-fatal diagnosis was based on the records of events obtained from hospitalization data. All cardiovascular events were independently censored by the end-point assessment committee, whose members were blinded to the baseline risk factor information of the study participants.

### Statistical Analysis

Data were presented as mean ± standard deviation (SD) and number (percentage). In addition, participants were classified based on BP and age group in order to evaluate the risk of developing cardiovascular events. Kaplan–Meier survival analysis was then used to estimate the risk of all-cause and CVD mortality, stroke, and MI, according to classification based on BP. The proportional hazard assumption was examined and met by plotting the log minus log survival curves. Moreover, Hazard ratios (HRs) for all-cause, CVD mortality, strokes, and MI in various categories of BP were calculated by Cox proportional hazards models, after adjusting for such factors as age, current smoking, current drinking, ethnicity, education level, physical activities, BMI, history of diabetes, family history of hypertension, history of hyperlipidemia at baseline. Participants with an SBP <120 mmHg and DBP <80 mmHg were considered as the controls. The Cox proportional hazards model was used to assess the interaction between age group and stage 1 hypertension in order to establish whether the risk of cardiovascular disease was heterogeneous among different age groups in participants with stage 1 hypertension. The proportional hazards assumption in the Cox proportional hazards model was affirmed using Schoenfeld residuals. All analyses were performed using IBM SPSS version 22.0 and STATA version 11.0 (Stata Corporation, College Station, TX, USA) and *P* < 0.001 was accepted as statistically significant.

## Results

**Figure 2:**
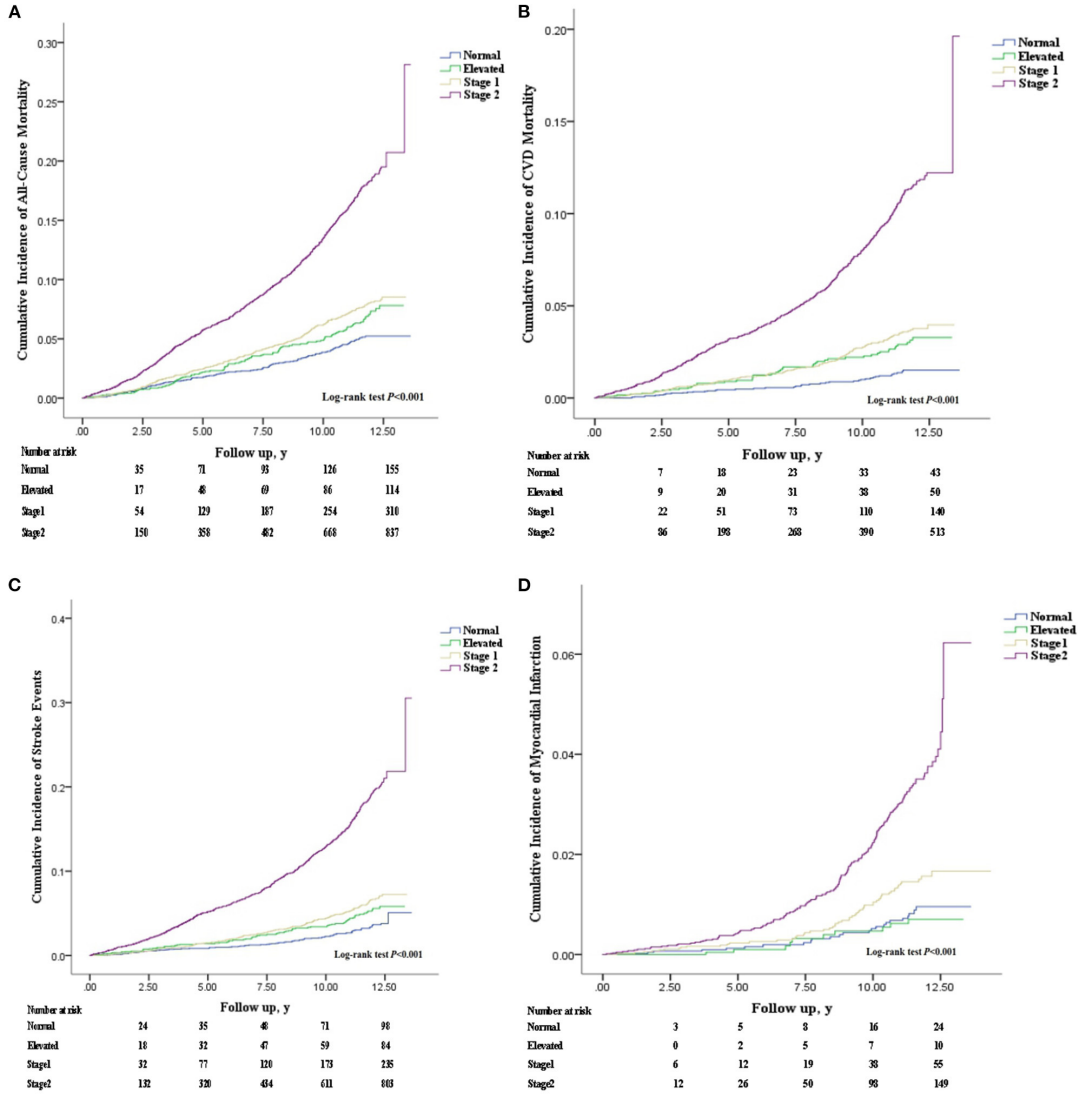
The cumulative incidence of adverse events by the different BP groups. Incident **(A)** All-Cause Mortality, **(B)** Cardiovascular Disease Mortality, **(C)** Stroke Incidence, and **(D)** Myocardial Infarction. Error bars represent 95% CI. Normal: SBP <120 mm Hg and DBP <80 mm Hg; Elevated: SBP of 120–129 mm Hg and DBP of <80 mmHg; Stage 1: SBP of 130–139 mmHg or DBP 80–89 mmHg; Stage 2: SBP/DBP ≥ 140/90 mm Hg, or taking antihypertensive medications. SBP, systolic blood pressure; DBP, diastolic blood pressure. CI, confidence interval. y, year; Stage 1, Stage 1 hypertension; Stage 2, Stage 2 hypertension.

Table 1 shows the baseline characteristics of the participants according to BP groups. In this study, the analysis involved 19,374 women aged ≥35 years. The results showed that the mean SBP and DBP were 132.3 ± 23.4 mmHg and 81.5 ± 12.6 mmHg, 29.5% of the participants had stage 1 hypertension. Differences were originated from different distribution of covariates in hypertensive cases with stage 1 hypertension compared to other groups including normal BP, elevated BP and stage 2 hypertension. Moreover, individuals with stage 1 hypertension appeared to be older, Mongolian, more hyperlipidemia, with lower degree of instruction, with higher BMI when compared with the normal BP. The respective cumulative incidence curves for adverse events in the BP categories, were generated through Kaplan–Meier survival analysis. The obtained results indicated that the cumulative incidence of all-cause mortality, CVD mortality, stroke, and MI increased progressively with elevated BP (*P* < 0.001). In particular, patients with stage 2 hypertension have the highest cumulative incidence of CVD mortality and stroke, followed by stage 1 hypertension, elevated BP, and normal BP (Figure 2).

**Table 1:** Baseline characteristics of study population (*n* = 19,374).

**Characteristics**	**Overall (*n*=19374)**	**Blood pressure groups**				
		**Normal**	**Elevated**	**Stage 1**	**Stage 2**	***P*-value**
		**(*n* = 4,437)**	**(*n* = 2,414)**	**(*n* = 5,724)**	**(*n* = 6,799)**	
Age, (years)	50.4 ± 11.5	46.0 ± 9.5	48.1 ± 10.5	49.1 ± 10.7	55.2 ± 12.0	<0.001
Current smoking, *n* (%)	3,064 (15.8)	616 (13.9)	330 (13.7)	785 (13.7)	1,333 (19.6)	<0.001
Current drinking, *n* (%)	1,178 (6.1)	220 (5.0)	119 (4.9)	288 (5.0)	551 (8.1)	<0.001
Ethnicity, *n* (%)						
Han	15,095 (77.9)	3,624 (81.7)	1,916 (79.4)	4,410 (77.0)	5,145 (75.7)	<0.001
Mongolian	4,026 (20.8)	755 (17.0)	466 (19.3)	1,239 (21.6)	1,566 (23.0)	
Other	253 (1.3)	58 (1.3)	32 (1.3)	75 (1.3)	88 (1.3)	
SBP, (mmHg)	132.3 (23.4)	108.2 ± 7.7	123.7 ± 3.0	126.6 ± 8.5	156.0 ± 21.7	<0.001
DBP, (mmHg)	81.5 (12.6)	69.4 ± 6.2	72.5 ± 5.3	81.1 ± 5.1	92.8 ± 12.0	<0.001
Education level, *n* (%)						
Primary school or below	9,728 (50.2)	1,718 (38.7)	1,075 (44.5)	2,719 (47.5)	4216 (62.0)	<0.001
Middle school	8,786 (45.3)	2,478 (55.8)	1,252 (51.9)	2,752(48.1)	2,304(33.9)	
High school or above	860 (4.4)	241 (5.4)	87 (3.6)	253 (4.4)	279 (4.1)	
Physical activities level, *n* (%)						
Low	5,854 (30.2)	950 (21.4)	693 (28.7)	1,582 (27.6)	2,629 (38.7)	<0.001
Medium	8,634 (44.6)	2,187 (49.3)	1,060 (43.9)	2,726 (47.6)	2,661 (39.1)	
Higher	4,886 (25.2)	1,300 (29.3)	661 (27.4)	1,416 (24.7)	1,509 (22.2)	
BMI						
<25	14,275 (73.7)	3,678 (82.9)	1,919 (79.5)	4236 (74.0)	4,442 (65.3)	<0.001
25–30	4,632 (23.9)	711 (16.0)	462 (19.1)	1,389 (24.3)	2,070 (30.4)	
>30	467 (2.4)	48 (1.1)	33 (1.4)	99 (1.7)	287 (4.2)	
History of diabetes, *n* (%)	85 (0.4)	13 (0.3)	6 (0.2)	17 (0.3)	49 (0.7)	<0.001
Family history of hypertension, *n* (%)	2,152 (11.1)	331 (7.5)	160 (6.6)	519 (9.1)	1,142 (16.8)	<0.001
History of hyperlipidemia, *n* (%)	491 (2.5)	35 (0.8)	32 (1.3)	93 (1.6)	331 (4.9)	<0.001

*Values are expressed as mean ± SD or n (%), BMI, body mass index, SBP, systolic blood pressure, DBP, diastolic blood pressure, Normal SBP <120 mmHg and DBP <80 mmHg, Elevated SBP 120–129 mmHg and DBP <80 mmHg, Stage 1 SBP 130–139 mmHg or DBP 80–89 mmHg, Stage 2 SBP ≥140 mmHg/DBP ≥90 mmHg or taking antihypertensive medications*.

**Figure 3:**
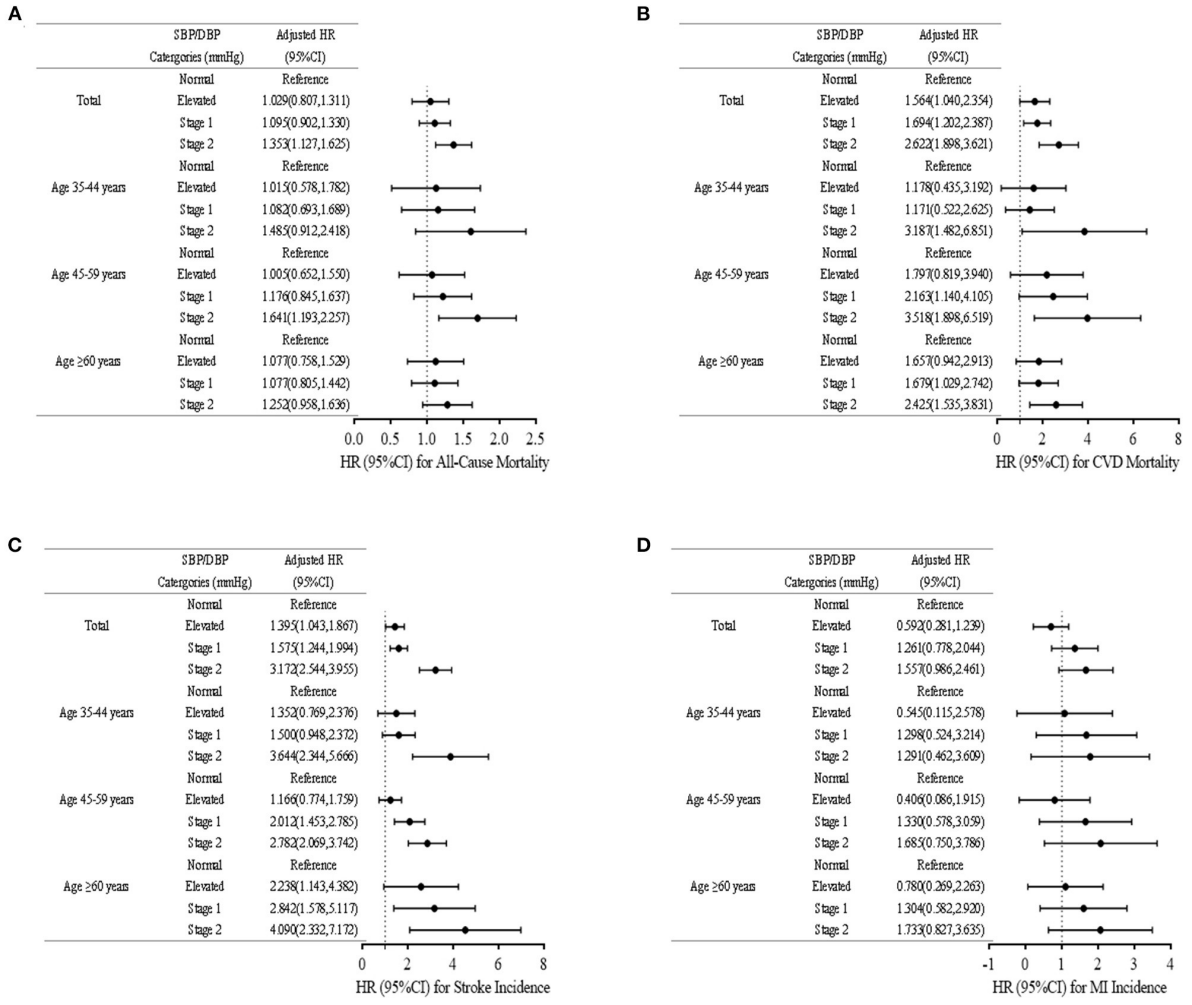
Cox regression analyses for association. Incident **(A)** All-Cause Mortality, **(B)** Cardiovascular Disease Mortality, **(C)** Stroke Incidence, and **(D)** Myocardial Infarction. Error bars represent 95% CI. Normal: SBP < 120 mm Hg and DBP < 80 mm Hg; Elevated: SBP of 120–129 mm Hg and DBP of <80 mmHg; Stage 1: SBP of 130–139 mmHg or DBP 80–89 mmHg; Stage 2: SBP/DBP ≥ 140/90 mm Hg, or taking antihypertensive medications. SBP, systolic blood pressure; DBP, diastolic blood pressure. CI, confidence interval. Adjusted for age, current smoking, current drinking, ethnicity, education level, physical activities, BMI, history of diabetes, family history of hypertension, history of hyperlipidemia.

For the present analysis, 1,419 of all mortalities were all cause-associated, 748 were CVD-associated, 1,224 were caused by stroke, and 241 were resulted from MI. We found that the incidence of cardiovascular events increased with rising baseline BP. In stage 1 hypertension the HR was 1.694 (95% CI: 1.202–2.387) in CVD mortality, and 1.575 (95% CI: 1.244–1.994) in stroke. The proportion of CVD mortality and stroke events were 17.1 and 21.6% in women aged ≥ 45 years, respectively. Compared with normal BP, stage 1 hypertension had a HR (95% CI) of 1.095 (0.902–1.330) for all-cause mortality, 1.694 (1.202–2.387) for CVD mortality, 1.575 (1.244–1.994) for stroke incidence, and 1.261 (0.778–2.044) for MI incidence. After adjusting for confounding factors, participants with stage 1 hypertension have a significantly higher risk of CVD mortality and stroke incidence compared to those with normal BP. The obtained results indicated that it accounted for 18.55% of CVD mortality and 23.96% of stroke incidence among rural women aged ≥ 45 years. Nonetheless, stage 1 hypertension was not associated with a higher risk of cardiovascular events in women aged between 35 and 44 years (Figure 3).

## Discussion

It is widely accepted that the risk of CVD in individuals who are in the same age group increases with an increase in BP. In addition, the risk of CVD increases differently in individuals from different age groups but have the same BP values. This study indicated that stage 1 hypertension was associated with a greatly increased risk of CVD mortality and incidence of stroke among rural women aged ≥45 years of Liaoning province, China, but not correlated with a higher risk of cardiovascular events in participants aged between 35 and 44 years, compared to normal BP. Moreover, stage 2 hypertension was also associated with a greatly increased risk of CVD mortality among women aged ≥45 years, and a greatly increased risk of stroke incidence among all age groups, compared to normal BP. These results therefore emphasize the importance of monitoring the BP values among rural women as it reduces the risk of cardiovascular events associated with hypertension. This is also one of the few prospective studies that have quantitatively evaluated the risk of cardiovascular disease among rural women based on the newly-defined guidelines for stage 1 hypertension. Therefore, these findings on the elevated risk of CVD linked to stage 1 hypertension will greatly inform efforts on the prevention of CVD (17).

Adoption of the 2017 ACC/AHA guidelines on hypertension will significantly increase the diagnosis of patients with the condition and ultimately require treatment, consequently reducing the risk of CVD. However, it is also controversial given that the risk of CVD is the main basis of determining the strategy for antihypertensive treatment. Several epidemiological studies have investigated the occurrence of adverse events attributable to stage 1 hypertension (18). Nonetheless, some of the studies limited the age of their study population to above 41 years, a cutoff that was not really representative of the entire population. In addition, subgroup analysis showed that stage 1 hypertension did not trigger a higher risk of CVD in participants aged above 60 years (19). Contrary to previously reported findings, the present study revealed that stage 1 hypertension participants aged ≥60 years were at greatly higher risk of cardiovascular events. However, the validity of these observations should be confirmed using a larger sample size. A previous study by Talaei et al. showed that the participants aged between 46 and 65 years with stage 1 hypertension were associated with an elevated risk of CVD-related deaths but with no history of CVD, compared to those normal BP (20). However, the study mainly focused on an aging population and the outcome did not include stroke and MI, thereby limiting the significance of the findings. The present study supplements previous research because subgroup analysis of participants aged 35 to 44 years showed no statistical difference in the increased risk of CVD associated with stage 1 hypertension. Furthermore, a previous study reported that high sodium and low potassium intake resulted in elevated blood pressure (21). It is worth noting that the participants in this study were mainly rural women from Northeast China, which is still among the regions with the highest levels of sodium intake in the world (22). The results indicated that salt intake was correlated with BP. Additionally, the high salt-intake population did to be higher prevalence of hypertension and average BP level.

The recent changes in the definition of stage 1 hypertension may affect disease management of the condition in China and even worldwide. Notably, Gabriel et al. reported that the association of stage 1 hypertension with CVD risk was stronger and more compelling in Western populations compared to Asian (23). Therefore, it is vital to establish clinical evidence in Asians because it was reported that there are racial differences between Asians and Westerners in the impact of blood pressure on major adverse cardiac events. Additionally, the consensus in many countries is that the new definition of stage 1 hypertension can enhance awareness on the risk of CVD, thereby improving vascular health, but individuals in low-income countries face more challenges than those in high-income countries when it comes to public health (24–26). The prevalence of stage 1 hypertension in developing countries is high but often underestimated due to its asymptomatic nature, thereby it seriously endangers human life and brings heavy economic burden to the society (27). According to previous research, the prevention and treatment of stage 1 hypertension in the rural areas of China should be improved because the current level of cognition, treatment, and control is low (28, 29). The present study incorporated a large sample size and a long follow-up period in order to increase the statistical power of the findings. It obtained herein also indicate stage 1 hypertension cause a significant increases in risk of CVD morbidity and mortality. In addition, more CVD events in rural Chinese women aged ≥45 years can be attributed to stage 1 hypertension. Similarly, a cohort study carried out by Linxian et al. that addressed stage 1 hypertension conferred a higher risk of cardiovascular events among rural Chinese aged 40–69 years, compared to individuals with normal BP (30). Therefore, it is expected that early detection of stage 1 hypertension and subsequent intervention will help in slowing down the progression of hypertension, thereby enhancing cardiovascular health, shielding against organ damage, and finally lowering the risk of developing CVD.

While this study uncovered some insightful findings, it had a few limitations. First, the cohort study involved a specific population of rural women form Northeast China, aged ≥35 years, it is not known whether the same conclusions can be applied to other ages, sex, or regions. A more diverse population is thus needed to confirm these findings. Second, the study lacked information on other potential confounders such as forms of secondary hypertension, blood biochemical data, and the absence of comorbidities, such as atrial fibrillation, obstructive sleep apnea, or other sleep disorders, that may have had a significant influence on the development of cardiovascular disease. Therefore, further research is required to explore the effect of the above mentioned factors. Third, some cases may not have been recorded due to the indistinct clinical symptoms and poor awareness on early medical intervention. The actual number of cases might therefore have been underestimated. Consequently, a more diverse population is required to confirm our findings.

## Conclusion

Our study mainly found that stage 1 hypertension based on the 2017 ACC/AHA guidelines was independently associated with an increased risk of CVD mortality and stroke among the rural northern Chinese women aged ≥45 years. The application of guidelines can be beneficial to reduce cardiovascular events through lowering BP in stage 1 population. Therefore, public health programs should closely monitor hypertension in rural women because its high prevalence may be accompanied by the risk of cardiovascular events.

## Data Availability Statement

The raw data supporting the conclusions of this article will be made available by the authors, without undue reservation.

## Ethics Statement

The studies involving human participants were reviewed and approved by Ethical clearance for the study was provided by the China Medical University Research Ethics Committee, reference No: 2014-2-2. Moreover, signed informed consent was freely obtained from all the participants involved in the study. The patients/participants provided their written informed consent to participate in this study. Written informed consent was obtained from the individual(s) for the publication of any potentially identifiable images or data included in this article.

## Author Contributions

JW was responsible for conceptualization, formal analysis, and writing the manuscript. WD and YJ were responsible for revising the manuscript, investigation, and data collection. LZ and SL contributed to mechanical testing, developed computational models, and analyzed data. ZS and YS were responsible for resources, supervision, and project administration. All authors have read and approved the final version of the manuscript.

## Conflict of Interest

The authors declare that the research was conducted in the absence of any commercial or financial relationships that could be construed as a potential conflict of interest.

## Publisher's Note

All claims expressed in this article are solely those of the authors and do not necessarily represent those of their affiliated organizations, or those of the publisher, the editors and the reviewers. Any product that may be evaluated in this article, or claim that may be made by its manufacturer, is not guaranteed or endorsed by the publisher.
