# Supplementary material for: The Association of Stage 1 Hypertension, Defined by the 2017 ACC/AHA Guidelines, With Cardiovascular Events Among Rural Women in Liaoning Province, China
Source: Front Cardiovasc Med. 2021 Aug 12;8:710500. doi: 10.3389/fcvm.2021.710500 (PMC8387632; doi:10.3389/fcvm.2021.710500)
Supplement: Supplementary file 1 [file Table_1.DOC]

Table 1

| Baseline characteristics of study population (*n*=19374) | | | | | | |
| --- | --- | --- | --- | --- | --- | --- |
| Characteristics | Overall (*n*=19374) | Blood pressure groups | | | | |
|  |  | Normal | Elevated | Stage 1 | Stage 2 | *P*-value |
|  |  | (*n*=4437) | (*n*=2414) | (*n*=5724) | (*n*=6799) |  |
| Age,(years) | 50.4±11.5 | 46.0±9.5 | 48.1±10.5 | 49.1±10.7 | 55.2±12.0 | <0.001 |
| Current smoking,*n*(%) | 3064(15.8) | 616(13.9) | 330(13.7) | 785(13.7) | 1333(19.6) | <0.001 |
| Current drinking,*n*(%) | 1178(6.1) | 220(5.0) | 119(4.9) | 288(5.0) | 551(8.1) | <0.001 |
| Ethnicity,*n*(%) |  |  |  |  |  |  |
| Han | 15095(77.9) | 3624(81.7) | 1916(79.4) | 4410(77.0) | 5145(75.7) | <0.001 |
| Mongolian | 4026(20.8) | 755(17.0) | 466(19.3) | 1239(21.6) | 1566(23.0) |  |
| Other | 253(1.3) | 58(1.3) | 32(1.3) | 75(1.3) | 88(1.3) |  |
| SBP,(mmHg) | 132.3(23.4) | 108.2±7.7 | 123.7±3.0 | 126.6±8.5 | 156.0±21.7 | <0.001 |
| DBP,(mmHg) | 81.5(12.6) | 69.4±6.2 | 72.5±5.3 | 81.1±5.1 | 92.8±12.0 | <0.001 |
| Education level,*n*(%) |  |  |  |  |  |  |
| Prinmary school or below | 9728(50.2) | 1718(38.7) | 1075(44.5) | 2719(47.5) | 4216(62.0) | <0.001 |
| Middle school | 8786(45.3) | 2478(55.8) | 1252(51.9) | 2752(48.1) | 2304(33.9) |  |
| High school or above | 860(4.4) | 241(5.4) | 87(3.6) | 253(4.4) | 279(4.1) |  |
| Physical activities level,*n*(%) |  |  |  |  |  |  |
| Low | 5854(30.2) | 950(21.4) | 693(28.7) | 1582(27.6) | 2629(38.7) | <0.001 |
| Medium | 8634(44.6) | 2187(49.3) | 1060(43.9) | 2726(47.6) | 2661(39.1) |  |
| Higher | 4886(25.2) | 1300(29.3) | 661(27.4) | 1416(24.7) | 1509(22.2) |  |
| BMI |  |  |  |  |  |  |
| <25 | 14275(73.7) | 3678(82.9) | 1919(79.5) | 4236(74.0) | 4442(65.3) | <0.001 |
| 25-30 | 4632(23.9) | 711(16.0) | 462(19.1) | 1389(24.3) | 2070(30.4) |  |
| >30 | 467(2.4) | 48(1.1) | 33(1.4) | 99(1.7) | 287(4.2) |  |
| History of diabetes,*n*(%) | 85(0.4) | 13(0.3) | 6(0.2) | 17(0.3) | 49(0.7) | <0.001 |
| Family history of hypertension,*n*(%) | 2152(11.1) | 331(7.5) | 160(6.6) | 519(9.1) | 1142(16.8) | <0.001 |
| History of hyperlipidemia,*n*(%) | 491(2.5) | 35(0.8) | 32(1.3) | 93(1.6) | 331(4.9) | <0.001 |
| (Values are expressed as mean ± SD or *n* (%), BMI body mass index, SBP systolic blood pressure, DBP diastolic blood pressure, Normal SBP <120 mmHg and DBP <80 mmHg, Elevated SBP 120–129 mmHg and DBP <80 mmHg, Stage 1 SBP 130–139 mmHg or DBP 80–89 mmHg, Stage 2 SBP ≥140 mmHg/DBP ≥90 mmHg or taking antihypertensive medications.) | | | | | | |
