# Supplementary material for: The Association of Stage 1 Hypertension, Defined by the 2017 ACC/AHA Guidelines, With Cardiovascular Events Among Rural Women in Liaoning Province, China
Source: Front Cardiovasc Med. 2021 Aug 12;8:710500. doi: 10.3389/fcvm.2021.710500 (PMC8387632; doi:10.3389/fcvm.2021.710500)
Supplement: Supplementary file 2 [file Table_2.DOC]

Supplementary tabulations:

Table 2

| Baseline characteristics of study population | | | |
| --- | --- | --- | --- |
| Characteristics | Blood pressure groups | | |
|  | Normal | Stage 1 | *P*-value |
|  | (n=4437) | (n=5724) |  |
| Age,(years) | 46.0±9.5 | 49.1±10.7 | <0.001 |
| Current smoking,n(%) | 616(13.9) | 785(13.7) | 0.806 |
| Current drinking,n(%) | 220(5.0) | 288(5.0) | 0.867 |
| Ethnicity,n(%) |  |  |  |
| Han | 3624(81.7) | 4410(77.0) | <0.001 |
| Mongolian | 755(17.0) | 1239(21.6) |  |
| Other | 58(1.3) | 75(1.3) |  |
| SBP,(mmHg) | 108.2±7.7 | 126.6±8.5 | <0.001 |
| DBP(mmHg) | 69.4±6.2 | 81.1±5.1 | <0.001 |
| Education level,n(%) |  |  |  |
| Prinmary school or below | 1718(38.7) | 2719(47.5) | <0.001 |
| Middle school | 2478(55.8) | 2752(48.1) |  |
| High schol or above | 241(5.4) | 253(4.4) |  |
| Physical activities level,n(%) |  |  |  |
| Low | 950(21.4) | 1582(27.6) | <0.001 |
| Medium | 2187(49.3) | 2726(47.6) |  |
| Higher | 1300(29.3) | 1416(24.7) |  |
| BMI |  |  |  |
| <25 | 3678(82.9) | 4236(74.0) | <0.001 |
| 25-30 | 711(16.0) | 1389(24.3) |  |
| >30 | 48(1.1) | 99(1.7) |  |
| History of diabetes,n(%) | 13(0.3) | 17(0.3) | 0.971 |
| Family history of hypertension,n(%) | 331(7.5) | 519(9.1) | 0.004 |
| History of hyperlipidemia,n(%) | 35(0.8) | 93(1.6) | <0.001 |
| (Values are expressed as mean ± SD or *n* (%), BMI body mass index, SBP systolic blood pressure, DBP diastolic blood pressure, Normal SBP <120 mmHg and DBP <80 mmHg, Stage 1 SBP 130–139 mmHg or DBP 80–89 mmHg.) | | | |
