# Supplementary material for: The Association of Stage 1 Hypertension, Defined by the 2017 ACC/AHA Guidelines, With Cardiovascular Events Among Rural Women in Liaoning Province, China
Source: Front Cardiovasc Med. 2021 Aug 12;8:710500. doi: 10.3389/fcvm.2021.710500 (PMC8387632; doi:10.3389/fcvm.2021.710500)
Supplement: Supplementary file 3 [file Table_3.DOC]

Supplementary tabulations:

Table 3

| Univariate analysis of covarities as association with Outcomes | | | | |
| --- | --- | --- | --- | --- |
| Characteristics | All-cause Mortality  HR(95%CI) | CVD Mortality  HR(95%CI) | Stroke  HR(95%CI) | MI  HR(95%CI) |
| Age,(years) | 1.105(1.100-1.110) | 1.121(1.115-1.128) | 1.058(1.054-1.063) | 1.103(1.092-1.115) |
| Current smoking,n(%) | 1.547(1.366-1.752) | 1.577(1.330-1.871) | 1.320(1.147-1.519) | 1.920(1.443-2.556) |
| Current drinking,n(%) | 1.324(1.092-1.604) | 1.335(1.025-1.738) | 1.231(0.994-1.524) | 1.768(1.168-2.675) |
| Ethnicity,n(%) |  |  |  |  |
| Han |  |  |  |  |
| Mongolian | 0.920(0.597-1.416) | 0.695(0.409-1.181) | 0.634(0.430-0.937) | 0.815(0.303-2.198) |
| Other | 0.938(0.603-1.460) | 0.834(0.484-1.436) | 0.653(0.436-0.977) | 0.952(0.345-2.624) |
| Systolic blood pressure(mmHg) | 1.022(1.020-1.024) | 1.028(1.026-1.031) | 1.026(1.024-1.028) | 1.023(1.019-1.028) |
| Diastolic blood pressure(mmHg) | 1.022(1.019-1.026) | 1.033(1.029-1.038) | 1.039(1.036-1.043) | 1.023(1.014-1.032) |
| Education level,n(%) |  |  |  |  |
| Prinmary school or below |  |  |  |  |
| Middle school | 4.132(2.734-6.244) | 6.575(3.273-13.208) | 1.858(1.348-2.562) | 3.132(1.288-7.615) |
| High schol or above | 1.162(0.760-1.777) | 1.437(0.703-2.938) | 0.845(0.607-1.176) | 0.928(0.370-2.326) |
| Physical activities level,n(%) |  |  |  |  |
| Low |  |  |  |  |
| Medium | 1.231(1.025-1.479) | 1.281(0.984-1.667) | 0.959(0.821-1.120) | 1.074(0.724-1.593) |
| Higher | 5.116(4.336-6.037） | 5.911(4.665-7.488) | 2.094(1.803-2.431) | 3.567(2.485-5.119) |
| Body mass index |  |  |  |  |
| <25 |  |  |  |  |
| 25-30 | 0.875(0.640-1.195) | 0.708(0.478-1.050) | 0.537(0.403-0.717) | 0.591(0.302-1.157) |
| >30 | 0.756(0.546-1.048) | 0.668(0.442-1.009) | 0.750(0.557-1.010) | 0.826(0.413-1.650) |
| Diabetes,n(%) | 3.470(2.232-5.396) | 2.980(1.545-5.751) | 3.869(2.429-6.163) | 2.091(0.520-8.411) |
| Family history of hypertension,n(%) | 0.583(0.476-0.714) | 0.543(0.408-0.724) | 1.074(0.905-1.274) | 0.868(0.574-1.314) |
| History of hyperlipidemia,n(%) | 1.287(0.968-1.711) | 1.392(0.955-2.031) | 2.385(1.880-3.027) | 1.550(0.823-2.919) |
| Follow-up SBP | 1.003(1.000,1.006) | 1.004(1.000,1.008) | 1.010(1.006,1.015) | 1.006(0.999,1.012) |
| Follow-up DBP | 0.999(0.994,1.004) | 0.998(0.991,1.005) | 1.003(0.995,1.011) | 1.006(0.994,1.018) |
